# A preliminary synopsis on amber scorpions with special reference to Burmite species: an extraordinary development of our knowledge in only 20 years

**DOI:** 10.3897/zookeys.600.8913

**Published:** 2016-06-22

**Authors:** Wilson R. Lourenço

**Affiliations:** 1 Muséum national d’Histoire naturelle, Sorbonne Université, Institut de Systématique, Evolution, Biodiversité (ISYEB), UMR7205-CNRS, MNHN, UPMC, EPHE, CP 53, 57 rue Cuvier, 75005 Paris, France

**Keywords:** Burmite, Cretaceous, fossil, Myanmar, new species, Palaeoburmesebuthidae, scorpion

## Abstract

A preliminary study on fossil scorpions found in amber, from the Lower Cretaceous through the Palaeocene and up to the Miocene is proposed. Scorpions remain rare among the arthropods found trapped in amber. Only 24 specimens are known from Cretaceous amber, representing eight families and subfamilies, ten genera and 21 species; in parallel, 10 specimens have been recorded from Baltic amber representing seven genera and ten species. A few more recent fossils from Dominican and Mexican amber have also been described. The present study of a new scorpion specimen from the Cretaceous amber of Myanmar (Burmite) resulted in the description of one new species, *Betaburmesebuthus bellus* **sp. n.** – belonging to the subfamily Palaeoburmesebuthinae Lourenço, 2015. The new description brings further elements to the clarification of the status of this subfamily, which is now raised to family level. Once again, this new Burmite element attests to the considerable degree of diversity in the Burmese amber-producing forests.

## Introduction

Among the fossil arthropods found in amber, scorpions remain extremely rare. The renewal of studies on scorpions trapped in amber began in the early 1980s when a few specimens from Dominican and Mexican amber were described (Schawaller 1979, 1982; Santiago-Blay and Poinar Jr. 1988, 1993; Santiago-Blay et al. 1990). Even if some new taxa from Dominican and Mexican amber are yet to be described, the amber fossils found in these regions of the world seem in all cases closely related to the extant scorpion taxa of the Caribbean and North/Central America.

Baltic amber was the first to provide fossil scorpions, at the beginning of the 19^th^ century. The first described species was *Scorpio schweiggeri* (Holl 1829). However, both the description and the illustration of this species lack accuracy; the only conclusion that can be reached is that the scorpion most certainly belongs to the family Buthidae C. L. Koch, 1837. This species has been ignored by most authors, although Schawaller (1979) published a brief comment in which he suggested that *Scorpio schweiggeri* should be considered a *nomen nudum*. Since the type-specimen was lost, there is not much that can be added on its status.

A second species, *Tityus eogenus* Menge, 1869, was also described. Unlike *Scorpio schweiggeri*, *Tityus eogenus* has received the attention of many authors, first because of its assignment by Menge to a typically Neotropical extant genus, and secondly because the type-specimen was apparently lost soon after its description, which prevented the confirmation of its taxonomic position. Therefore this Baltic amber scorpion has turned into a kind of curiosity. Because of the early disappearance of Menge’s material, for more than one hundred years this Baltic amber fossil has been the subject of discussion and speculation, and was mentioned in a number of publications (e. g. Werner 1935; Petrunkevitch 1955, 1958; Larsson 1978; Schawaller 1979; Kjellesvig-Waering 1986; Spahr 1993). Menge’s collection included two specimens, but apparently only one was preserved well enough to be of scientific value (Menge 1869; Larsson 1978). Based on the characters supplied by Menge (1869), it can only be concluded that *Tityus eogenus* is indeed a buthid scorpion. It could, however, be assigned equally well to any of several genera within this family.

In 1995, a new specimen of scorpion from Baltic amber was located in Hamburg, Germany. After the examination of all visible characters it was determinated as a member of the family Buthidae, belonging to a new genus and a new species, allied to the genus *Lychas* C. L. Koch, 1845. Nothing, however, could clearly associate this specimen to the two species previously described by Holl (1829) and Menge (1869). The main findings of these studies demonstrated that this Baltic amber scorpion could be associated with the Old World extant fauna. Several new discoveries followed and were subject of a number of studies since 1996 (Lourenço 2004, 2012a; Lourenço and Weitschat 1996, 2000, 2001, 2005, 2009; Lourenço et al. 2005). This led to discovering and describing a total of ten specimens representing seven new genera and ten new species, globally confirming the relationships of this extinct fauna with the elements of the extant buthid fauna yet present in both the old and new worlds.

Even more relevant was the description, in the last 15 years, of 24 specimens from Cretaceous amber, representing eight families and subfamilies, ten genera and 21 species. These fossil scorpions trapped in Cretaceous amber can be dated from 135 to 90 Ma. Although several of these elements can be associated with buthoids, such as *Archaeobuthus estephani* Lourenço, 2001 (family Archaeobuthidae Lourenço, 2001) from amber of Lebanon and the several species of the genera *Palaeoburmesebuthus* Lourenço, 2002 and *Betaburmesebuthus* Lourenço, 2015 (subfamily Palaeoburmesebuthinae Lourenço, 2015) both from Burmite amber from Myanmar (Lourenço and Beigel 2015), a number of non-buthoid elements have also been recorded and described. These comprise *Palaeoeuscorpius gallicus* Lourenço, 2003 (family Palaeoeuscorpiidae Lourenço, 2003) from amber of France and several elements from Burmite, namely: *Electrochaerilus buckleyi* Santiago-Blay, Fet, Soleglad & Anderson, 2004 (family Chaerilidae Pocock, 1893), several species of the genus *Chaerilobuthus* Lourenço & Beigel, 2011 (family Chaerilobuthidae Lourenço & Beigel, 2011), *Palaeotrilineatus ellenbergeri* Lourenço, 2012 (family Palaeotrilineatidae Lourenço, 2012), *Archaeoscorpiops cretacicus* Lourenço, 2015 and *Burmesescorpiops groehni* Lourenço, 2016 (subfamily Archaeoscorpiopinae Lourenço, 2015), and finally *Sucinlourencous adrianae* Rossi, 2015 (family Sucinlourencoidae Rossi, 2015). Dated at almost 135 Ma, *Archaeobuthus estephani* remains the oldest fossil scorpion ever found in amber (Lourenço 2001, 2002, 2003, 2012b, 2013, 2015a,b,c,d, 2016; Lourenço and Beigel 2011; Lourenço and Velten 2015, 2016; Rossi 2015; Santiago-Blay et al. 2004).

After the clarification of the familial status of the genus *Palaeoburmesebuthus* (and consequently of the genus *Betaburmesebuthus*) and its placement in the subfamily Palaeoburmesebuthinae, this last subfamily was temporarily accommodated in the family Archaeobuthidae Lourenço, 2001, both because of their association to the buthoid lineage, but in particular because of their location in a similar geological horizon. Nevertheless, the recent study of several almost perfectly preserved specimens, clearly attests their relationship to the buthoids (Lourenço 2015b, c; Lourenço and Velten 2016; this study), in particular based on their trichobothrial patterns, which are almost identical to those of several extant buthoids. Based on these new characters (see description), the subfamily Palaeoburmesebuthinae is now raised to the familial level as Palaeoburmesebuthidae and placed in the superfamily Buthoidea.

### The controversial opinions of different authors concerning the presence of buthoid lineages during Mesozoic times

The exclusion of the family Archaeobuthidae from the buthoids has been proposed by a number of authors (Baptista et al. 2006; Riquelme et al. 2015), mainly on the basis of theoretical speculation. It is well known that both higher classification of scorpion in general and the classification of fossils in particular are controversial issues, which have been largely debated within scorpion taxonomy, especially during the last 20 years. There is no full evidence allowing the inclusion or the exclusion of a given fossil to the Buthoidea lineage.

This new procedure is becoming a common method used by many authors in their attempt to gain credit on the backs of other people’s discoveries. In a few cases only the originally described material has been re-examined, but in many other cases the critics were based solely on the originally published descriptions (see Riquelme et al. 2015). In fact, when dealing with systematic studies of a given taxonomic group, it should be a standard practice to refer to and consider the available material cited in previous publications. Moreover, since the available fossil specimens are extremely rare, this paucity of information makes the re-evaluation even more challenging; consequently, it remains unclear to what extent the re-evaluations on the same case specimens are of any value.

In the case of *Archaeobuthus estephani* Lourenço, 2001 (family Archaeobuthidae) all available data are based only on a single but incomplete specimen. The validity of the family Archaeobuthidae was not questioned of itself, but authors such as Baptista et al. (2006) clearly rejected its association to the buthoids. Nevertheless, the available data to date for this unique Lebanon amber fossil is still insufficient to proceed with a revision of the position of this taxon. Therefore, a final decision should await until more information becomes available.

In a recent publication, eight authors have described a new fossil scorpion from Chiapas amber (Riquelme et al. 2015). If this new fossil is considered as rare and outstanding by its authors, it should be noted that not only is Chiapas amber of recent Miocene age, some 20-15 Ma, but also that other species have been recently described from this same type of amber (Lourenço 2014), bringing as much, if not more, morphological information about these fossils. In the case of the species described by Riquelme et al. (2015), some key morphological information such as the trichobothrial pattern is not clearly defined and in all cases not illustrated, attesting that the described specimen was likely not so unique. The major problem, however, with this publication is the fact that the authors rapidly diverge from the main goal, which should have been the thorough description of the new fossil scorpion, and instead proposed a type of global synopsis on scorpion fossils in amber, including comments on sedimentary fossils and even copal sub-fossils. All these extensive sections presented in part in the introduction, comments, and the phylogenetic discussion are not supported by any original data, but remain solely a compilation of data already available in several original or second-hand publications. Furthermore, the presented compilation, including the phylogenetic analyses, is conducted without any new study of the original material, which most certainly remains totally unknown to these authors. Even more controversial is the attempt to produce a synopsis on amber scorpions, thereby ignoring many of the currently known elements. A number of gaps are clearly visible, in particular concerning Cretaceous Burmite amber scorpions. The most critical mistake, however, is the proposition in a suggested cladogram (cf. figure 10 in Riquelme et al. 2015) of the Burmite genus *Palaeoburmesebuthus* as *incertae sedis*, knowing that the familial status of this genus was already clarified several months before their publication (Lourenço 2015a). See systematic section for further discussion.

## Material and methods

The specimen investigated here is preserved in very clear block of pale yellow amber. Details of the block are supplied together with the description of the specimen. Many characters, and in particular several trichobothria, are visible in this specimen, allowing detailed investigation. Some characters, however, are not totally observable mainly because the specimen suffered a certain degree of dissection process within the resin. The schematic drawings provided here are interpretations of what was observable. Illustrations and measurements were produced with the aid of a Wild M5 stereomicroscope equipped with a drawing tube and an ocular micrometer. Measurements follow Stahnke (1970) and are given in mm. Trichobothrial notations follow Vachon (1974). Trichobothria were definitely recorded only when their bothria (areoles) could be observed. Other trichobothria may be suggested by the presence of transverse hairs.

## Systematic description

### Superfamily Buthoidea C. L. Koch, 1837

#### Family Palaeoburmesebuthidae

Taxon classificationAnimaliaScorpionesPalaeoburmesebuthidae

Lourenço, 2015 stat. n.

##### Diagnosis for the family.

General morphology shows similarities with several elements of extant buthid scorpions. The following combination of features can be used to diagnose the new family (see also Lourenço 2015a): Carapace not granulated, smooth; anterior margin with a moderately marked median concavity, as observed in some extant buthids. chelicerae with moderately long distal teeth which do not clearly overlap; fixed and movable fingers with one basal and one median tooth. Vesicle very long, with a pear-like shape, resembling those of some extant buthids; with a very long aculeus. Fixed and movable fingers of pedipalp chela with a series of small rounded granules, without any conspicuous accessory granules. Trichobothrial pattern with elements ressembling those of extant buthid type A (Vachon 1974); dorsal trichobothria of femur disposed in alpha (α) or beta (β) configurations (see Vachon 1975). Tibial spurs present on legs III and IV.

##### Type species.

*Palaeoburmesebuthus grimaldii* Lourenço, 2002

#### Genus *Betaburmesebuthus* Lourenço, 2015

##### Betaburmesebuthus bellus sp. n.

Taxon classificationAnimaliaScorpionesPalaeoburmesebuthidae

http://zoobank.org/21267812-28A4-4D1A-BD96-A2D71ACC11F8

Figures 1–4 , 5–7 , 8–9 , 10

###### Holotype.

A juvenile, most certainly a male. Very clear block of pale yellow amber that measured 21.5 × 17.0 × 2 mm. Only a few inclusions and bubbles prevent a 100% observation of all characters. Type locality and horizon: Myanmar (Burma), Kachin; precise locality unknown; Lower Cretaceous.

###### Etymology.

The specific name is an epythet from Latin “*bellus*” meaning beautiful, elegant.

###### Repository.

The type specimen is deposited in the collection of the Museum of the Geological-Palaeontological Institut of the University of Hamburg (CeNak - Centrum of Natural History), Coll. N° 4586, Coll. Gröhn N° 11086.

###### Diagnosis.

General morphology is globally similar with other species of the genus *Betaburmesebuthus* Lourenço, 2015 and also with extant buthoid scorpions. The following combination of features can be used to diagnose the new species: Carapace weakly to moderately granulated; anterior margin with a moderately marked median concavity. Sternum subpentagonal. Tergites with three carinae, the lateral totally inconspicuous. Sternites with small oval to slit-like spiracle. Metasomal segments I and II with ten carinae; setation on all metasomal segments strongly marked. Fixed and movable fingers of pedipalp chela with a series of small rounded granules and no conspicuous spinoid accessory granules. Trichobothrial pattern with elements ressembling those of extant buthid type A (Vachon 1974): at least 1-3 internal, five dorsal, and two external trichobothria on the femur; dorsal trichobothria disposed in beta (β) configuration (Vachon 1975); one internal and five dorsal trichobothria on patella; 6-7 external trichobothria can be suggested on patella by the presence of fine setae; some are displaced on ventral aspect; 4-5 dorso-external and two ventral on chelal hand; six on fixed finger. Tibial spurs present on legs III and IV.

###### Description.

Coloration: the scorpion is yellow to slightly reddish-yellow; carapace and tergites yellow; metasomal segments yellow; telson reddish-yellow; pedipalps and legs yellow. Ventral aspect yellow; coxapophysis slightly darker than dorsal aspect.

###### Morphology.

Carapace weakly granular; anterior margin with a moderately marked median concavity. Carinae inconspicuous; furrows weak. Median ocular tubercle clearly anterior to the centre of carapace; median eyes moderate in size and separated by more than one ocular diameter. Three pairs of lateral eyes of moderate size. Sternum subpentagonal. Mesosomal tergites weakly granular, with one median carina; lateral carinae totally inconspicuous; VII with five weakly marked carinae. Pectines large, with 18-17 teeth; fulcra absent. Sternites weakly granular to smooth, with small oval to slit-like spiracles. Metasomal segment I to IV rounded with 10-10-8-8 carinae; segment V slender with five carinae; dorsal carinae of segments I-IV with minute spinoid granules; dorsal aspect of segments I to V weakly depressed; setation on all segments strongly marked. Telson with a very long pear-shaped vesicle; weakly granular to smooth; aculeus extremely long and moderately curved; setation strongly marked. Cheliceral dentition only partially visible; fixed and movable fingers with one basal tooth observable; distal teeth moderately long (Vachon 1963). Pedipalp femur pentacarinate; patella with 6-7 carinae; internal face of femur and patella with spinoid granules. Chela with weakly marked carinae; all faces not granular, smooth. Fixed and movable fingers each with one series of small rounded granules and no conspicuous spinoid accessory granules; extremity of fingers with stronger spinoid granules; setation of pedipalps weakly marked. Trichobothriotaxy recalling type A (Vachon 1974) of extant buthids: at least 1-3 internal, five dorsal, and two external trichobothria on the femur; dorsal trichobothria disposed in beta (β) configuration (Vachon 1975); one internal, five dorsal, and no ventral trichobothria on patella; 6-7 external trichobothria can be suggested on patella by the presence of fine setae; some are displaced on the ventral aspect 5-6 dorso-external and two ventral on chelal hand; six on fixed finger. Tibial spurs present on legs III and IV, moderately marked.

Morphometric values (in mm) of male juvenile holotype of *Betaburmesebuthus bellus* sp. n.

Total length 11.80 (including telson). Carapace: length 1.40, anterior width 0.87, posterior width 1.40. Mesosoma length 3.60. Metasomal segments. I: length 0.74, width 0.60; II: length 0.84, width 0.60; III: length 0.94, depth 0.57; IV: length 1.14, depth 0.50; V: length 1.67, depth 0.47. Telson length 1.47. Vesicle: depth 0.37. Pedipalp: femur length 1.14, width 0.34; patella length 1.24, width 0.47; chela length 2.24, width 0.34; movable finger length 1.57.

**Figures 1–4.**
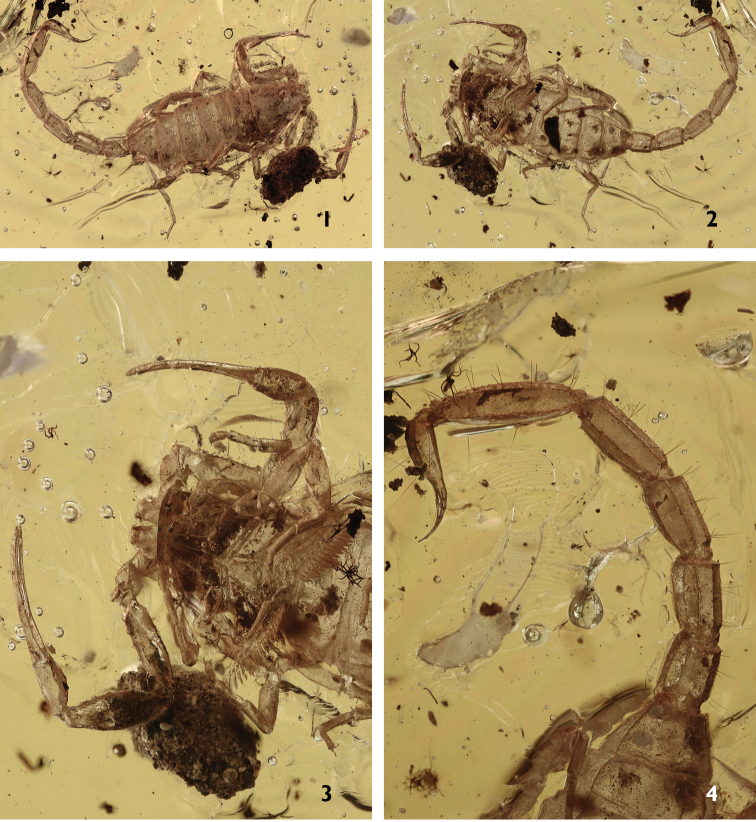
*Betaburmesebuthus bellus* sp. n. Male holotype. **1–2** Habitus, dorsal and ventral aspects **3** Ventral aspect in detail, showing sternum, genital operculum and pectines **4** Tergites VI-VII, metasomal segments and telson, dorso-lateral aspect.

**Figures 5–7.**
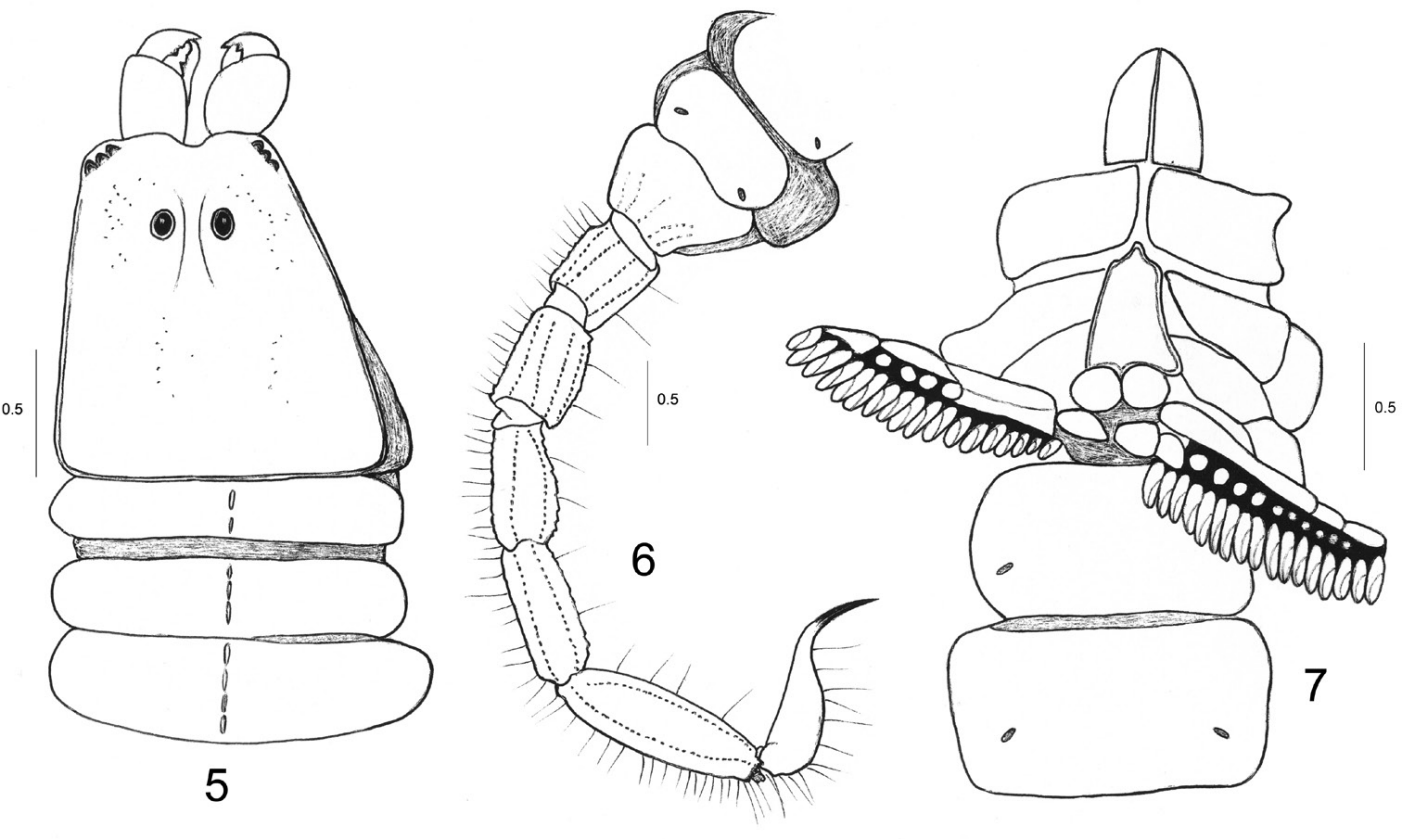
*Betaburmesebuthus bellus* sp. n. Male holotype. **5** Chelicera, carapace and tergites I-III, dorsal aspect **6** Sternites V-VII showing carinae and spiracles and metasomal segments I-V and telson, ventro-lateral aspect **7** Ventral aspect, showing Coxapophysis, sternum, genital operculum, pectines and sternites with spiracles. Scale bars: 0.5 mm.

**Figures 8–9.**
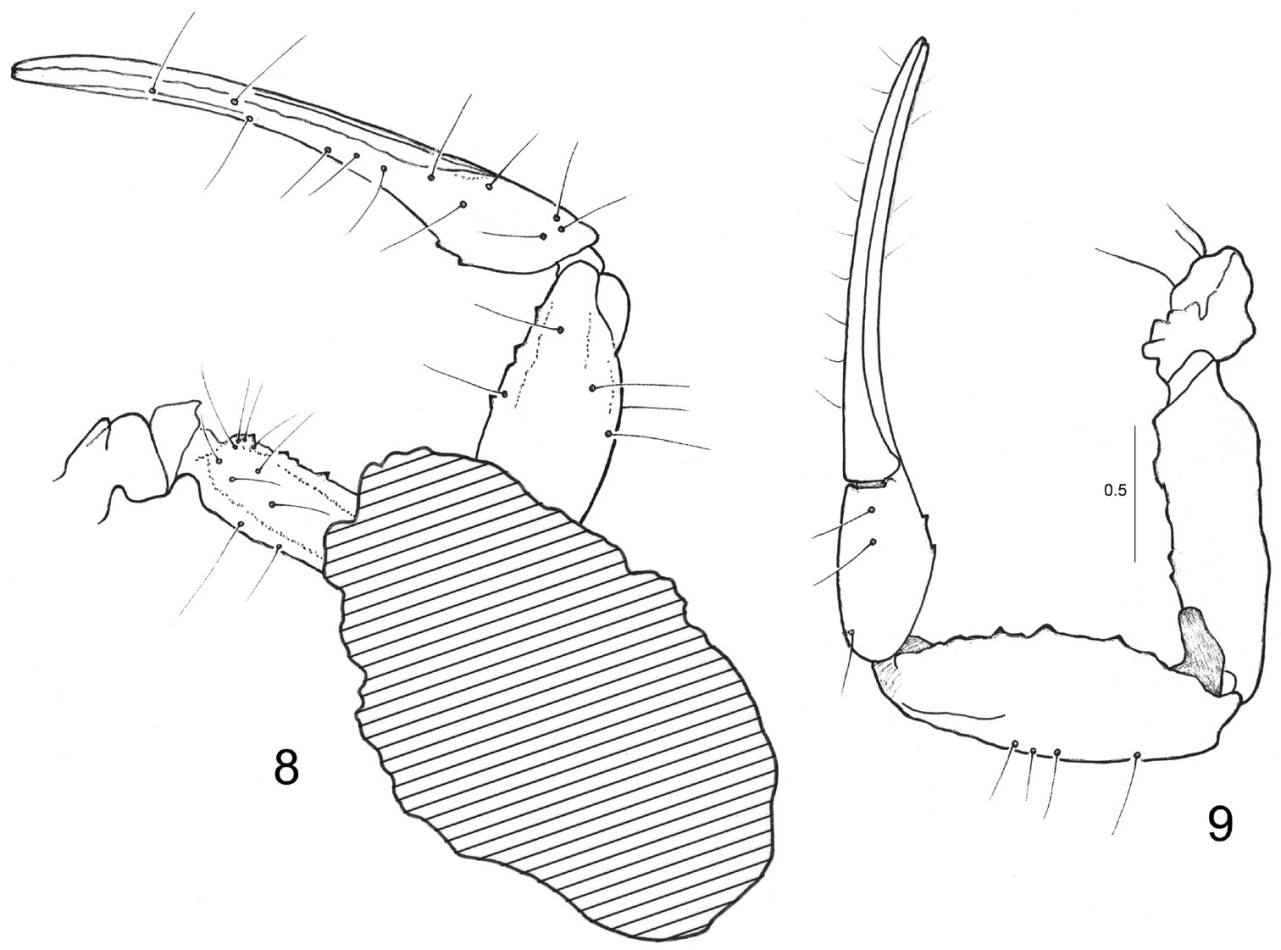
*Betaburmesebuthus bellus* sp. n. Male holotype. Righ pedipalp, dorsal and ventral aspects, showing trichobothrial pattern. Dorsal aspect is partially covered by an inclusion. Scale bar: 0.5 mm.

**Figure 10.**
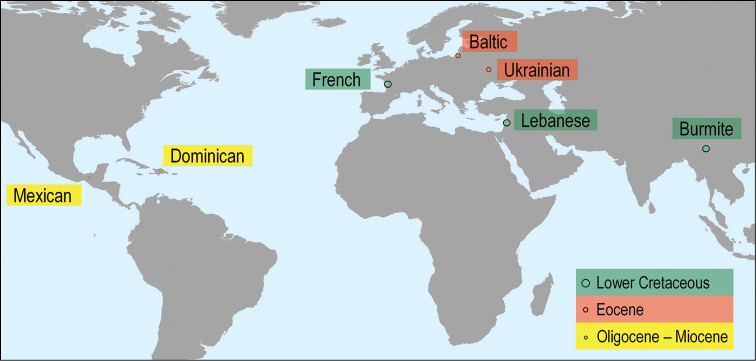
World map showing the sites where scorpions included in amber have been found.

#### Key to the species of Palaeoburmesebuthidae

**Table:** 

1	Dorsal trichobothria of pedipalp femur in alpha disposition	***Palaeoburmesebuthus*- 2**
–	Dorsal trichobothria of pedipalp femur in beta disposition	***Betaburmesebuthus*- 3**
2	Telson aculeus moderately long and sharp; metasomal segments III-V with moderately marked carinae	***Palaeoburmesebuthus grimaldii***
–	Telson aculeus extremely long and sharp; metasomal segments III-V with strongly marked carinae	***Palaeoburmesebuthus ohlhoffi***
3	Internal face of pedipalp patella without any or to a maximum one spinoid tubercle	**4**
–	Internal face of pedipalp patella with two or four strong spinoid tubercles	**6**
4	Spiracles rounded	**5**
–	Spiracles slit-like	***Betaburmesebuthus bellus* sp. n.**
5	Pectines with 20-20 teeth	***Betaburmesebuthus kobberti***
–	Pectines with 14-15 teeth	***Betaburmesebuthus muelleri***
6	Internal face of pedipalp patella with two strong spinoid tubercles; two pairs of lateral eyes	***Betaburmesebuthus bidentatus***
–	Internal face of pedipalp patella with four strong spinoid tubercles; three pairs of lateral eyes	***Betaburmesebuthus fleissneri***

## Supplementary Material

XML Treatment for Palaeoburmesebuthidae

XML Treatment for Betaburmesebuthus bellus
